# In vitro antileishmanial activities of hydro-methanolic crude extracts and solvent fractions of *Clematis simensis fresen* leaf, and *Euphorbia abyssinica* latex

**DOI:** 10.1097/MD.0000000000038039

**Published:** 2024-05-03

**Authors:** Kassahun Misgana Worku, Dawit Araya, Habtie Tesfa, Eshetie Melese Birru, Asrat Hailu, Mulugeta Aemero

**Affiliations:** a Department of Medical Laboratory Science, College of Medicine and Health Sciences, Arba Minch University, Arba Minch, Ethiopia; b Department of Microbiology, Immunology and Parasitology, Faculty of Medicine, College of Health Sciences, Addis Ababa University, Addis Ababa, Ethiopia; c Department of Medical Parasitology, School of Biomedical and Laboratory Sciences, College of Medicine and Health Sciences, University of Gondar, Gondar, Ethiopia; d Department of Pharmacology, School of Pharmacy, College of Medicine and Health Sciences, University of Gondar, Gondar, Ethiopia.

**Keywords:** antileishmanial, hemolytic, in vitro, medicinal plants, phytochemical

## Abstract

As a result of increasing drug resistance, crossover resistance development, prolonged therapy, and the absence of different agents with innovative methods for implementation, the efficacy of recent antileishmanial medications is severely declining. So, it is vital to look for other medications from botanical remedies that have antileishmanial activity. The latex of *Euphorbia abyssinica (E abyssinica*) and the leaves of Clematis simensis fresen (*C simensis*) were macerated in methanol (80%). In vitro antileishmanial activity of the preparation was tried on promastigotes of Leishmania aethiopica (*L aethiopica*) and *Leishmania donovani (L donovani*) using resazurin assay, and fluorescence intensity was measured. One percent of dimethyl sulfoxide (DMSO) and media as negative control and amphotericin B as positive control were used. Additionally, hemolytic & phytochemical tests of the preparation were done. The mean and standard errors of each extract were evaluated and interpreted for statistical significance using one-way analysis of variance. From sigmoidal dose-response curves of % inhibition, half maximal inhibitory concentration (IC_50_) values were determined by GraphPad Prism and Microsoft Excel; outcomes were presented as mean ± standard error of mean of triplicate trials. *P* < .05 was statistical significance. The phytochemical screening of *C simensis* and *E abyssinica* confirmed the existence of steroids, phenols, tannins, saponins, alkaloids, terpenoids, flavonoids and glycosides. *C simensis* possesses antileishmanial activity with IC_50_ outcomes of 46.12 ± 0.03 and 8.18 ± 0.10 µg/mL on the promastigotes of *L aethiopica* and *L donovani*, respectively. However, *E abyssinica* showed stronger activity with IC_50_ outcomes of 16.07 ± 0.05 µg/mL and 4.82 ± 0.07 µg/mL on *L aethiopica* and *L donovani*, respectively. *C simensis* and *E abyssinica* have a less hemolytic effect on human red blood cells at low concentrations. The outcomes from this investigation demonstrated that the preparation of *C simensis* and *E abyssinica* indicated significant antileishmanial activity. Therefore, further in vivo assessment of antileishmanial, cytotoxicity activity and quantitative identification of secondary metabolites are highly recommended.

## 1. Introduction

Leishmaniasis is an obligate intracellular protozoan disease spread by the bite of female blood-sucking sand fly the genus Phlebotomine.^[1]^ This is classified as a neglected tropical disease (NTD) which is established in different areas of the world such as South Europe, the tropics, and the subtropics.^[2]^ Based on the nature of clinical symptoms, leishmaniasis has 3 major types; Visceral leishmaniasis (VL), Mucocutaneous leishmaniasis (MCL) and Cutaneous leishmaniasis (CL): these are highly prevalent in developing countries related to malnutrition, poor housing, low income, weak immunity, and population displacement.^[1]^

*Leishmania infantum* and *Leishmania donovani (L donovani*) are the primary causes of VL. Exceptionally, dermatotropic *Leishmania* species can induce VL in some immunosuppressed patients.^[3]^ A serious medical disease called clinical VL that goes untreated could be fatal. Infections are mainly managed by a reaction of granulomatous tissues that is self-limiting; however, it may not fully eradicate parasites.^[4]^ Mucosal (or mucocutaneous) leishmaniasis (MCL) is characterized by mucosal infection or ulceration of mucous membranes.^[5]^ A skin condition known as CL is brought on by several *Leishmania* parasites.^[6]^ Papules, nodules, and ulcers that heal with scarring are present in the lesions, and lymphadenitis can be detected, particularly in CL produced by the *vianna* sub-genus.^[7]^

Extra 12 million individuals are infected with leishmaniasis, making it one of the top 10 NTDs worldwide.^[8]^ Every year, 700,000 to 1 million more cases are thought to occur (50,000–90,000 for VL and 0.6 million–1 million for CL) and 20,000 to 30,000 fatalities worldwide.^[9,10]^ High morbidity, mortality, and quality of life issues are all caused by leishmaniasis, along with stigma, psychosocial morbidity, and mental illness.^[11]^ Leishmaniasis care is very expensive compared to other illnesses, ranging from US$ 30 to US$ 1500 (USD) for medications alone, and this is considered a significant contributor to the poverty of affected individuals. Moreover, toxicity and resistance to chemotherapy are increasing.^[12]^

Leishmaniasis has few available treatment options. Because the majority of patients come from underdeveloped nations, drugs for this disease are not an appealing target for the income-oriented therapeutic sector to devote investigation and advance resources.^[13]^ To cure leishmaniasis, chemotherapeutic medicines are employed, including pentavalent antimonials,^[14]^ amphotericin B formulations, miltefosine,^[15]^ paromomycin^[16]^ and pentamidine isethionate.^[17]^ CL is also treated with physical therapy, which uses cryotherapy and thermotherapy.^[18]^

In impoverished nations like Ethiopia, leishmaniasis is a main public health issue with a pooled prevalence of 9.13%. This is due in part to malnutrition as a result of poverty, weak immunity, and the disease inability to respond to prescribed medications.^[19]^ Drug development is mostly disregarded because of the inability of users to pay for such treatments.^[20,21]^ The efficacy of present antileishmanial medicines is drastically decreasing due to rising resistance to medication, developing tolerance, needs for parenteral administration and/or extent of therapy, an absence of novel medications with new mechanisms of action, and the absence of efficient vaccinations.^[22,23]^ As a result, innovative, economical, safe, effective and easily administered medications are desperately needed.^[24]^

Medicinal plants could be a source of novel medications in the future.^[25]^ As a result of availability and biomedical safety, there is a great scale of practice and concern in medicinal herbs in Ethiopia.^[26]^ This is because natural yields have long been known to be noble bases of pharmacologically active substances that can help with a variety of ailments, including leishmaniasis.^[27–30]^ Among these plants, the fresh leaves of *Clematis simensis fresen (C simensis*)^[31–35]^ and latex of *Euphorbia abyssinica (E abyssinica*)^[32,36,37]^ are widely used by traditional healers to treat leishmaniasis. Besides to rumors on the practice of fresh leaves of *C simensis* and latex of *E abyssinica* in folk medicines, plants also contain different secondary metabolites.^[38–42]^

Despite the antileishmanial claims of *C simensis* and *E abyssinica*, to date, there has been no information in the studies on the antileishmanial action of the plants. The current investigation was therefore designed to assess the antileishmanial activity of hydro-methanolic crude preparation and solvent fractions of the leaf of *C simensis*, and latex of *E abyssinica* efficacy used as *Leishmania* management in Ethiopian traditional healing.

## 2. Materials and methods

### 2.1. Ethics approval

The School of Biomedical and Laboratory Sciences, College of Medicine and Health Sciences, University of Gondar Ethical Review Committee granted its approval for the study, which was then carried out (Reference number: SBMLS/2790/2021). A support letter was obtained from the Department of Medical Parasitology. Informed consent was not given due to the absence of involvement of patients.

### 2.2. Plant material

Fresh leaves of *C simensis* and latex of *E abyssinica* were collected from Gondar City, which is found in the Amhara National Regional State, North West Ethiopia. In May 2021, *E abyssinica* was obtained by breaking open sections of the bark on its stems and branches. A jar was attached to the bottom of the latex-dripping aperture. Similarly, *C simensis* was carefully collected and separated from other morphological parts of the herb. It was washed away with clean tap water to get rid of dust particles and other water-soluble impurities that settled on it.

The plants were validated by Mr. Abiyu Enyew, a botanist in the Department of Biology, University of Gondar. The numbers were assigned to each plant, *C simensis* KMW001/2021 and *E abyssinica* KMW002/2021, and stored in the botanical collections of the Department of Biology for future reference.

### 2.3. Chemicals and reagents

Chemical and reagents used in the study include hexane (Alpha Lab. Ltd., India), ethyl acetate (Alpha lab. Ltd., India), absolute methanol (Taflen Industries, Ethiopia), chloroform (Carlo Erba, Franc), distilled water (Leishmaniasis Research and Diagnostic Laboratory (LRDL), Addis Ababa University), H_2_SO_4_ (Blulux Laboratories, India), lead acetate (Guangdong Guanghua chemicals), potassium iodide (Calibre Engineering), HCl (Pentokay Laboratories), ammonia (Blulux Laboratories, India), Ferric Chloride (India), Wagner reagent (Fin chem. industries), dimethyl sulfoxide (laborchemikalien GmbH, Germany), D (+) glucose (anhydrous) (Sigma-Aldrich), heat-inactivated new born calf serum (HINBCS), phosphate buffer saline (PBS) (Gibco), nutrient agar (Sigma-Aldrich), calcium chloride (Sigma-Aldrich), potassium chloride (Sigma-Aldrich), sodium bicarbonate (Sigma-Aldrich), resazurin sodium salt (Sigma-Aldrich, Germany), sodium chloride (Sigma-Aldrich), Triton X-114 (Sigma-Aldrich Laborchemikaien GmbH, Germany), roswell park memorial institute-1640 (RPMI-1640) (Sigma-Aldrich, UK), and penicillin-streptomycin solution (Sigma-Aldrich, Co., MO).

### 2.4. Instruments and apparatuses

The instruments and apparatuses used in the study include: Drying oven (Abron Instruments, India), Whatman filter paper No1 (Maidstone, UK), gauze (Nylon clothes), Magnetic stirrer, vortex (whirl VIB2), Separatory funnels, Round bottom flasks (England), polystyrene sterile tissue culture flasks (Corning incorporated), Rotary evaporator (Yamato, Japan), Digital analytical balance (India), Lyophilizer (Love freeze instruments group Ltd, Germany), Deep freezer, table-top vacuum pump (India), Water bath (Yamato, Japan), Mortar and Pestle, Olympus inverted type light microscope (Shinjuku, Tokyo, Japan), Micro pipettes (Pipetman ultra), multichannel pipettes (Hamilton), Micro pipettes (Pipetman ultra), pipette tips and its rack (Eppendrof), Eppendorf tube (Corning incorporated), 96 well microtiter plates (Nalge Nunc international), Biological safety cabinet class IIA (Laboculture ESCO 903), Victor3 Multilabel Counter (PerkinElmer, Waltham, MA), and Wallac 1420 (Perkin/Elmer Corp., Germany).

### 2.5. Leishmania parasite strains and reference drug

Stored samples of *Leishmania aethiopica (L aethiopica*) (311/17) and *L donovani* (Gr/990) were taken from LRDL, in the Department of Microbiology, Immunology and Parasitology, School of Medicine, College of Health Sciences, Addis Ababa University. All antileishmanial assays were performed at LRDL, School of Medicine, College of Health Sciences, Addis Ababa University. Amphotericin B (Laborchemikaien GmbH, Germany), supplied by LRDL, at Department of Microbiology, Immunology and Parasitology, School of Medicine, College of Health Sciences, Addis Ababa University, Ethiopia was used as a positive control.

### 2.6. Extraction and fractionation

The crude extraction with 80% methanol was carried out according to Birhan et al, 2018.^[41]^ A total of 250 g of *C simensis* powder and 291 g of *E abyssinica* powder were macerated in 1500 mL and 1750 mL of 80% methanol, respectively, in Round bottom flasks were shaken occasionally for 3 consecutive nights. Surgical gauze and Whatman filter paper No. 1 were used in sequence to filter the extracts on day 3. The marc was doubly macerated in a similar way, and the filtrates that remained were then gathered and evaporated using a rotary evaporator set at 40°C, leaving behind a concentrated aqueous solution. A dry oven set to 40°C was used to additionally evaporate the leftover concentrated aqueous solution. The concentrated filtrate was placed in a deep freezer and frozen for an entire night at −40°C. The frozen filtrate was then lyophilized in order to remove the water at a vacuum pressure of 0.200 mBar at −40°C. The powder that had dried was measured, sealed in a jar, and kept at 4°C. For *C simensis* and *E abyssinica*, the yields following extraction of the dry powder material were 39 g (15.6%) and 53 g (18.2%), respectively.

According to Birhan et al (2018), fractionation was carried out using non-polar solvents.^[41]^ A separatory funnel was used to suspend 30 g of the crude extract in 250 mL of distilled water for fractionation. Hexane was then added in an amount equal to that, stirred thoroughly, and then allowed to settle and separate into layers. The hexane portion was then separated by releasing the bottom aqueous layer from the separatory funnel after each of the different layers had formed. The hexane fractions were collected in identical containers after repeating this procedure 3 times. Equal parts of ethyl acetate and distilled water were added to the aqueous fraction in order to create ethyl acetate portions. After a distinct layer between the ethyl acetate and the aqueous fraction had developed, the portion containing ethyl acetate was separated by removing the bottom aqueous component. The ethyl acetate fractions were collected in a similar container after this procedure was carried out twice more. The separated portions of all hexane and ethyl acetate were evaporated with a rotary evaporator. The aqueous portion was concentrated in an oven at 40°C before being lyophilized at a vacuum pressure of 0.200 mBar and −40°C. The percentage yield of the dry portion was estimated and the fractions were placed in sealed bottles and kept in the refrigerator at 4°C till needed.

### 2.7. Preliminary phytochemical screening

Qualitative phytochemical analyses were performed on 80% methanol crude extract and solvent fractions. Screening was done using standard phytochemical reagents for the existence or nonexistence of secondary metabolites like alkaloids (Wagner test), flavonoids (Shinoda test), terpenoids and steroids (Salkowski test), phenols and tannins (Ferric Chloride test), saponins (Foam test), anthraquinones (Borntranger test) and glycosides (Keller-Killiani test) following standard procedures.^[42]^

### 2.8. Leishmania culture

The parasites *L aethiopica* and *L donovani* were cultivated in Lock treated NovyMacNeal-Nicolle (NNN) medium holding antibiotic solution (penicillin 100 IU/mL and streptomycin 100 µg/mL). The logarithmic phase parasites were relocated from NNN media to tissue culture flasks having a complete RPMI-1640 medium (RPMI-1640 medium added with 10% HINBCS and 100 IU penicillin/mL-100 µg/mL streptomycin solution) at 22°C for *L aethiopica* and 26°C for *L donovani*. To guarantee the parasites’ optimal development, the culture was checked daily for 2 weeks. For an antipromastigote test, the parasites’ logarithmic phase was utilized.^[43,44]^

### 2.9. Antipromastigote assay

Every plant preparation was added to the initial well of a separate 96-well microtiter plate having 100 µL of complete culture medium to obtain an ending concentration of 100 µg/mL. Then, 100 µL was taken into the following wells, and the final 100 µL was cast off, to achieve 2-fold serial dilution. Next, 100 µL suspensions of parasites from earlier cultures were put into each well, each of which contained 3.5 × 10^6^ promastigotes of *L aethiopica* or *L donovani*. After 68 hours of incubation at 22°C, 20 µL (One-tenth of every single well overall volume) of resazurin (0.125 mg/mL) was placed and shielded with aluminum foil and allowed to rest for 4 hours. After an overall 72 hours of incubation, the level of fluorescence was assessed using the Victor3 Multilabel Counter at 544 nm for excitation and 590 nm for emission. The fluorescent properties of resazurin when introduced into drug-treated cultures were used to study the antileishmanial effect of the test samples. The experiment was carried out in threefold and contrasted with the reference medication (AMB), and negative controls (1% dimethyl sulfoxide [DMSO] and medium alone). Cell viability was tracked throughout the assay by monitoring the fluorescent signal. The total amount of live cells correlated with the fluorescence intensities.^[45,46]^ In this study, the level of in vitro activity of reference remedies, the crude extract and its solvent fractions were accepted according to the next criteria: half maximal inhibitory concentration (IC_50_) < 10 µg/mL, good activity; IC_50_ 10 to 50 µg/mL, moderate activity; IC_50_ 50 to 100 µg/mL, low activity; and IC_50_ > 100 µg/mL, inactive.^[47]^ Accordingly, the crude extract and solvent fractions were exposed to antileishmanial activity tests using the promastigote stages of *L aethiopica* and *L donovani*.

### 2.10. In vitro hemolytic activity

Red blood cells (RBCs) obtained from O + human whole blood (2 mL) from the blood bank at Tikur Anbessa Specialized Hospital were placed in 48 mL of PBS and centrifuged at 3500 rpm for 10 minutes at 4°C to measure lysing capacity. Following 3 PBS washes of the supernatant, about 1 ml of RBC pellets were formed.^[48]^ In order to create a 2% blood solution, the resultant pellet was re-suspended in 49 mL of PBS, and the concentration was adjusted to 1.9 × 109 RBC/mL. Subsequently, to create the total volume of 1500 mL, 200 mL of the blood solution was pipetted into Eppendorf tubes holding each test item at concentrations of 3.13, 6.25, 12.5, 25, 50, and 100 g/mL.^[49]^ Accurately prepared solutions with 2.5 × 10^8^ RBC/mL were incubated at 37°C for 2 hours. The ability of each test substance to rupture RBC cell membranes and release hemoglobin into the solution was used to establish how effective it was in destabilizing membranes. Centrifuging the mixture at 3500 rpm for 10 minutes caused the mixture to remain intact and burst RBC to pellet, releasing the hemoglobin in the floating liquid. In 96-well plates, 75 µL of the supernatant from each tube was obtained, and the absorbance was determined at 540 nm using a Wallac 1420. As a positive control, 50 µL of blood was added to 100 µL of Triton X-114, and the mixture was left to stand at 37°C for 30 minutes.^[50]^ As a negative control, RBC preparation containing 1% DMSO was applied. The hemolytic effects of each test plant were expressed as a percentage according to the following formula:


$$\begin{array}{l} \;\%\;\;\;Hemolysis=\left(\frac{At-An}{Ac-An}\right)*100 \end{array}$$

Where: A_t_ is the absorbance of the test sample.

A_n_ is the absorbance of the negative control (1% DMSO)

A_c_ is the absorbance of the positive control (Triton X-114)

### 2.11. Data analysis

The mean and standard errors of each treatment group were evaluated and interpreted for statistical significance using a one-way analysis of variance. Utilizing the computer program GraphPad Prism 8.4.3, antileishmanial activity (IC50) values were computed with sigmoidal dose-response curves of percentage inhibition and Microsoft Excel; values were expressed as mean ± standard error of mean of triplicate experiments. *P* < .05 was statistical significance.

## 3. Results

### 3.1. The yield of crude extracts and solvent fractions

A total of 250 and 291 grams of dried leaf from *C simensis* and latex of *E abyssinica* were harvested, respectively. The crude extract provides 39 grams of *C simensis* and 53 grams of *E abyssinica*. The percentage yield for the extracts of *C simensis* leaf, and *E abyssinica* latex in hexane, ethyl acetate, methanol, and aqueous as solvents in order of increasing polarity. Aqueous portions of both *C simensis* leaf and *E abyssinica* latex gave the highest yield (40% and 53.3%, respectively), while the hexane extract gave the lowest yield (3.3% and 6.66%, respectively) (Table 1).

**Table 1:** Yields of 80% methanol crude extract and solvent fractions of *C simensis* leaf and *E abyssinica* latex.

Plants	Yield (%)			
	Hexane	Ethyl acetate	Methanol (Crude)	Aqueous
*C simensis*	3.3	13.3	15.6	40
*E abyssinica*	6.66	10	18.2	53.3

### 3.2. Preliminary phytochemical screening

The results of phytochemical studies on ethyl acetate, hexane, aqueous, and methanol extracts of *C simensis* leaf and *E abyssinica* latex are presented in Table 2. These assays demonstrate the existence of some secondary metabolites, which are thought to be crucial for their therapeutic properties.

**Table 2:** Phytochemical screening of leaf of *C simensis* and latex of *E abyssinica*.

Secondary metabolites	Test used	*C simensis*				*E abyssinica*			
		Methanol	Hexane	Eth. Acetate	Aqueous	Methanol	Hexane	Eth. acetate	Aqueous
Alkaloids	Wagner test	+	+	−	+	+	+	+	+
Terpenoids	Salkowski test	+	+	−	−	−	−	−	−
Flavonoids	Shinoda test	+	−	+	+	−	−	−	−
Tannins	Ferric chloride test	+	−	+	+	−	−	−	−
Saponins	Foam test	+	−	−	+	+	−	−	+
Phenols	Ferric chloride test	+	−	+	+	+	−	+	+
Anthraquinones	Bontrager test	−	−	−	−	−	−	−	−
Steroidscom	Salkowski test	−	−	−	−	+	+	+	−
Glycosides	Keller-killani test	−	−	−	−	+	−	+	+

[+] indicates the presence of phytochemicals; [−] Indicates the absence of phytochemicals in the extract

Phenols, saponins, tannins, flavonoids, alkaloids, and terpenoids were detected in methanol extract, while alkaloids and terpenoids were found in hexane extract, according to the outcomes of the phytochemical screening of *C simensis* leaf. The aqueous portion, instead, contained phenols, saponins, tannins, flavonoids, and alkaloids, while the ethyl acetate extract contained tannins, flavonoids, and phenols. Most phytochemicals were found to be present in both methanol, and aqueous extract than the hexane, and ethyl acetate (Table 2).

In the case of *E abyssinica,* latex revealed that the methanol extract showed the presence of alkaloids, saponins, phenols, steroids, and glycosides. Whereas, the hexane portion revealed the existence of alkaloids and steroids. The ethyl acetate portion showed the presence of alkaloids, phenols, steroids, and glycosides, while the aqueous extract showed alkaloids, saponins, phenols, and glycosides (Table 2).

### 3.3. In vitro antileishmanial activity

IC_50_ (effective concentration required to achieve 50% growth inhibition) values for *C simensis* and latex of *E abyssinica* were determined against promastigotes of *L aethiopica* and *L donovani* in vitro. As shown in Table 3, the extracts tested showed antileishmanial action on the promastigote stages of *L aethiopica* and *L donovani* with IC_50_ values extending from 2.10 to 46.12 µg/mL. *E abyssinica* was suggestively (*P* < .0001) more active than *C simensis*. The present study shows that the leaf of *C simensis* crude extract possesses antileishmanial activity with IC_50_ values of 46.12 ± 0.03 and 8.18 ± 0.10 µg/mL on the promastigotes stage of *L aethiopica* and *L donovani*, respectively. Furthermore, the latex of the crude extract of *E abyssinica* showed antileishmanial activity at a lower concentration compared to the leaf of *C simensis* against both promastigotes of *L aethiopica* and *L donovani* with IC_50_ values of 16.07 ± 0.05 µg/mL and 4.82 ± 0.07µg/mL, respectively.

**Table 3:** The antileishmanial activity of leaf of *C simensis* and latex of *E abyssinica*.

Test samples/drugs	Antileishmanial activity IC_50_ (µg/mL)*	
	*Leishmania aethiopica*	*Leishmania donovani*
*C simensis*		
Crude (Methanol)	46.12 ± 0.03	8.18 ± 0.10
Aqueous	27.16 ± 0.08	5.76 ± 0.08
Ethyl Acetate	3.30 ± 0.12	25.71 ± 0.08
Hexane	3.47 ± 0.09	6.60 ± 0.09
*E abyssinica*		
Crude (Methanol)	16.07 ± 0.05	4.82 ± 0.07
Aqueous	2.10 ± 0.03	23.68 ± 0.11
Ethyl Acetate	3.91 ± 0.09	30.84 ± 0.06
Hexane	4.45 ± 0.13	15.29 ± 0.06
Amphotericin B	4.13 ± 0.04	5.75 ± 0.094
Media alone (NC)	0.00†	0.00†
1% DMSO (NC)	0.00†	0.00†

Values expressed as mean *± *SEM (n = 3); NC: Negative control; IC_50_: Effective concentration required to achieve 50% growth inhibition in µg/mL.

DMSO = dimethyl sulfoxide.

* Effective concentration required to achieve 50% growth inhibition in µg/mL.

† No effect.

The ethyl acetate fraction of *C simensis* leaf showed the lowest IC_50_ (3.30 ± 0.12 µg/mL) against *L aethiopica* when it is compared to hexane and aqueous fractions. Similarly, its aqueous fraction showed a lower IC_50_ value (5.76 ± 0.08 µg/mL) against *L donovani* promastigotes. In contrast, the aqueous fraction of *E abyssinica* showed the lowest IC_50_ value (2.10 ± 0.03 µg/mL) against *L aethiopica* when compared with other solvent fractions and the hexane fraction showed the lowest IC_50_ value (15.29 ± 0.06 µg/mL) against *L donovani*. Similar to the crude extract, the latex of *E abyssinica* solvent fractions antileishmanial activity at a lower concentration when compared to that of the leaf of *C simensis* against both promastigotes of *L aethiopica* and *L donovani* with IC_50_ values of 2.10 ± 0.03 µg/mL for aqueous fraction and 15.29 ± 0.06 µg/mL for hexane fraction, respectively.

### 3.4. In vitro hemolytic activity

**Figure 1.**
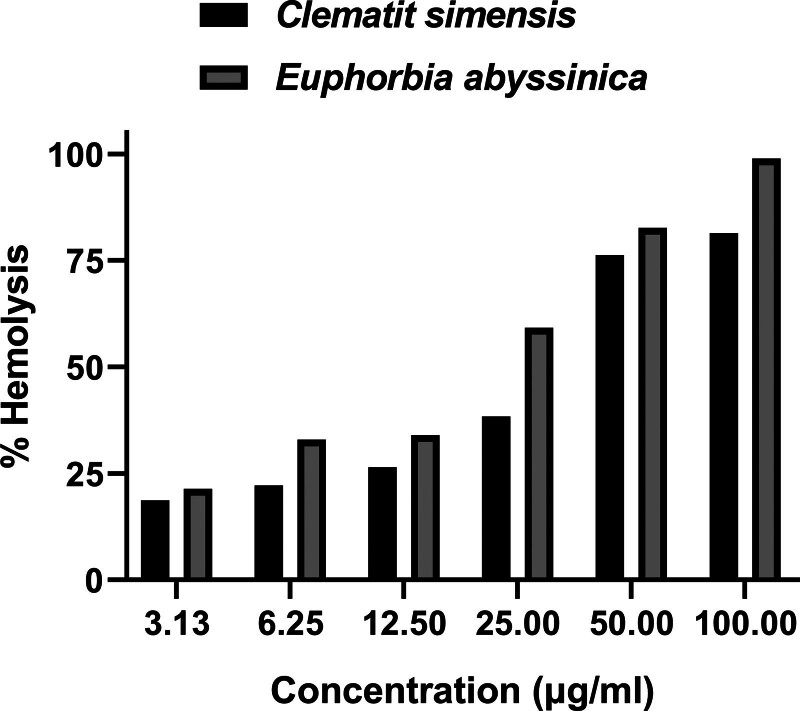
Hemolytic activity of the leaf of *C simensis* and latex of *E abyssinica* against human RBCs. RBC = red blood cell.

Hemolytic action of the crude preparation (80% methanol) of the *C simensis* leaf and latex from *E abyssinica* was tested against healthy human RBC. The preparations exhibited low to mild hemolytic effects on human RBCs. The hemolytic results of individual trial plants were stated as a percentage as shown in Figure 1. The result indicated that the *C simensis* leaf extract has low hemolytic activity when compared to the *E abyssinica* latex extract. The extracts showed a dose-dependent increase in hemolytic activity.

## 4. Discussion

Leishmaniasis is a severe and frequently fatal NTD that mostly affects people in the poorest countries and is linked to immune system weakness, population displacement, substandard housing, and malnutrition.^[1]^ In poor nations like Ethiopia, leishmaniasis is a serious medical issue with increased unresponsiveness of the disease to suggested drugs.^[19]^

The pharmacological activity of therapeutic plant extracts includes antibacterial, antioxidant, anticancer, anti-inflammatory, and antileishmanial properties; these actions are attributed to a range of biologically active functional components.^[29,30,51]^ According to previous studies, test plants have antioxidant,^[41]^ antibacterial,^[38,41,52,53]^ antifungal,^[40]^ antimalarial,^[39,42]^ and anticancer^[54]^ activity. *C simensis* and *E abyssinica* are commonly used by locals and users of indigenous medicine in Ethiopia to treat leishmaniasis.^[31,32,34–37]^

The extraction was accomplished through the use of the maceration process of *C simensis* leaf and *E abyssinica* latex which yielded a high percentage of (80%) methanol crude preparation. The solvent fractionation produced a greater percentage for the aqueous fraction than the other solvent fractions. The yield of plant extracts varied significantly in the current investigation. Because each plant has a different chemical makeup, some medicinal plants might have active ingredients that are more soluble in organic solvents while others may include substances that are more dissolved in water, which could account for the variation in the yield of aqueous and other solvent extracts of herbal remedies.^[55]^ Differences in the pharmacological characteristics of plants, and these in turn affect the percentage yield of preparations from several of the same plant species, are caused by the components of the plants used, the amount of time of the plant, the season, and the region of collection.^[56]^

According to the findings of the current investigation, both the crude preparations and the solvent fractions of *C simensis* leaf contain different types of phytochemicals depending on the types of solvents used. Alkaloids, terpenoids, flavonoids, tannins, saponins, and phenols were identified in the crude preparation of the *C simensis* leaf. The aqueous fraction contains compounds similar to the crude extract except for the terpenoids. While ethyl acetate portion contains flavonoids, tannins, and phenols, the hexane portion contains alkaloids and terpenoids. Preliminary phytochemical screening conducted previously in the Enarj Enawga district showed the existence of tannins, polyphenols, flavonoids, and terpenoids in 80% methanol preparation of *C simensis*,^[41]^ which is consistent with the current study. Another study from Gondar City,^[42]^ on 80% methanol extract of *C simensis* root confirmed the occurrence of secondary metabolites like alkaloids, terpenoids, flavonoids, and phenols, which is consistent with the current findings. The findings of this study are also in agreement with research from Bale, which shows the presence of flavonoids, tannins, and polyphenols from methanol extracts of *C simensis* leaf.^[57]^

Similarly, the current study findings indicated that *E abyssinica* latex contains different types of phytochemicals. The crude extract was positive for qualitative phytochemicals of alkaloids, saponins, phenols, steroids, and glycosides whereas the aqueous fraction includes similar phytochemical components as the crude extract except steroids. The ethyl acetate fraction contains the same phytochemicals as the crude extract except saponins. Hexane fractions contain only alkaloids and steroids. A previous study from the Kiremu district in Ethiopia on the bark extract of *E abyssinica* indicates the presence of tannins, flavonoids, phenols, carbohydrates, saponins, and steroids.^[58]^ Another study from the Somali region of Ethiopia on ethanolic and methanolic preparation of *E abyssinica* stem indicated the presence of alkaloids, flavonoids, phenolics, saponins, terpenoids, and tannins.^[38]^ Methanolic extracts of the same genus revealed the presence of phytosterols, saponins, alkaloids, flavonoids, and phenols that have been shown to be effective on *Leishmania* parasites.^[59]^

The difference in the kinds and concentrations of the phytochemical components might be due to the variation in ingredient solubility between diluents. A substance different solubility might rely on the solvents’ physical and chemical characteristics as well as the components of plants. The types, quantities, and relations of secondary metabolites that existed in crude extract and solvent fractions are contributing factors of antileishmanial activity.^[60]^

The current study discovered that the leaf of *C simensis* and latex of *E abyssinica* hydro-methanolic crude extract possess moderate and good antileishmanial activity on the promastigotes stage of *L aethiopica* and *L donovani*, correspondingly. *E abyssinica* was identified to be significantly (*P* < .0001) more active than *C simensis* when demonstrated by its IC_50_ value. This is in line with previous investigation on hydro-methanolic crude extract of the genus *Euphorbia* with antileishmanial effects with IC_50_ < 15.6 µg/mL.^[59]^ This might be because of secondary metabolites’ existence such as glycosides and steroids which are in favor of antileishmanial effect.^[61,62]^ The activity of solvent fractions, ethyl acetate, and hexane fractions of *C simensis* leaf shows good activity against *L aethiopica* when compared with aqueous fractions with moderate activity. Similarly, aqueous and hexane fractions of *C simensis* were more active on the promastigote stage of *L donovani,* which is in line with earlier studies done on the antileishmanial actions of the same genus from Costa Rica and South Africa. The dichloromethane and methanolic (IC_50_ = 10.5 and 55.3 µg/mL, respectively) extracts of *C. brachiata* showed a promising antileishmanial effect against *L donovani* promastigote, and the ethanolic extract of *C. dioica* possesses an IC_50_ of 27.5 µg/mL.^[63,64]^ This antileishmanial effect might be due to the presence of secondary metabolites with an antileishmanial effect such as flavonoids and phenols.^[65,66]^

All *E abyssinica* latex fractions (aqueous, hexane, and ethyl acetate) have noble action against *L aethiopica* and moderate action against *L donovani* promastigotes. The ethyl acetate and hexane fractions of *C simensis,* besides the aqueous and ethyl acetate fractions of *E abyssinica,* were more active on the promastigote stage of *L aethiopica* and had a better effect than the reference drug amphotericin B. The results of the current investigations are in line with a study from Morocco on the compounds of *E. resinifera* and *E. officinarum* methanolic extracts and hexane fraction of *E peplus* from a similar genus with significant antileishmanial activity.^[67]^ The aqueous fraction of *E abyssinica* revealed the highest antileishmanial activity while the crude preparation of *C simensis* had the least effect on *L aethiopica* promastigote. Instead, the crude preparation and the ethyl acetate preparation of *E abyssinica* possess the highest and least effects on *L donovani* promastigotes, respectively. Overall, the aqueous fraction of *E abyssinica* is the most effective and that of crude extract of *C simensis* is the least effective one.

When the antileishmanial activity of the extract of these plants contrary to promastigote forms of the 2 species were matched with regular antileishmanial medications, the solvent fraction of both *C simensis* and *E abyssinica*, the hexane portion of *C simensis,* and the aqueous portion of *E abyssinica* showed better activity than the reference drug on *L aethiopica* promastigotes, while the crude extract of *E abyssinica* was more active than the reference drug on *L donovani* promastigotes. Hexane and aqueous fractions of *C simensis* revealed the highest antileishmanial activity on *L aethiopica* and *L donovani*, respectively. Similarly, the aqueous fraction and crude extract of *E abyssinica* had the highest antileishmanial activity on *L aethiopica* and *L donovani*, respectively. An assessment of this information with the detected IC_50_ values of the reference drug (good antileishmanial activity on *L aethiopica* and *L donovani,* respectively) suggested that the 2 compounds indicated activity comparable to the reference drug Could be considered for additional activity tests.

Compared to *L donovani*, the majority of the extracts demonstrated relatively stronger activity against promastigote of *L aethiopica*. Modest variations in the metabolic properties and membrane composition of the 2 species, as well as the degree to which they are revealed to various compounds and cultural settings, could contribute to these variances in drug sensitivity between the 2 strains.^[68–70]^

Thus, the antileishmanial activity of herbal products, which are abundant in different phytochemicals, possibly results from the particular elements and/or their synergistic interplay within the primary chemicals and the additional effects that could be attributable to plant preparation minor elements.^[51]^ Results of the investigation suggested that the antileishmanial action of the crude extract and solvent fractions of *C simensis* leaf and *E abyssinica* latex were attributed to the existence of secondary compounds like steroids, glycosides, alkaloids, terpenoids, flavonoids, tannins, saponins, and phenols.

Natural substances originating from herbs have drawn a lot of interest because of their capacity to function as cytotoxic and chemopreventive agents. To ascertain whether a specific drug has antioxidant, antimicrobial, and other biological actions that could be exploited in therapeutic uses, a hemolytic assay is carried out.^[71]^ It is also helpful to determine whether the cytotoxic activity is caused by actual harm to the cell membrane or not. The hemolytic activity of any chemical is a sign of an overall cytotoxicity to normal healthy cells.^[72]^

In the present study, the toxicity of both the hydro-methanolic crude extract of the leaf of *C simensis* and the latex of *E abyssinica* was tested for their toxicity by their hemolytic actions in vitro. The hemolytic activity of the herbs’ preparations was matched using the Triton X-114, taken as a positive control, showing 100% hemolysis, and 1% DMSO, taken as a negative control, having 0% hemolysis. It was discovered that an increase in dose caused the hemolytic percentage to rise. This could be as a result of the presence of saponin in the herbal preparations, which is responsible for the hemolysis of erythrocytes.^[73]^ This will be an initial study on the hemolytic activity of *C simensis* leaves and *E abyssinica* latex.

## 5. Conclusion

In the current investigation, preliminary bioassay testing was done on 80% methanol extract and solvent fractions of the leaf of *C simensis* and the latex of *E abyssinica* showed the effectiveness of the plants as an antileishmanial agent against *L aethiopica* and *L donovani*. The aqueous fraction of *E abyssinica* is the most effective, and that of the crude extract of *C simensis* is the least effective. Further preliminary phytochemical study of the preparation leads to the isolation of essential secondary metabolites for instance flavonoids, alkaloids, tannins, terpenoids, saponins, glycosides, steroids, and phenols. The in vitro hemolytic test indicated that the leaf of *C simensis* and the latex of *E abyssinica* showed a dose-dependent increase in hemolysis of erythrocytes. The present investigation has offered scholarly support for the actual action of *C simensis* and *E abyssinica* against *Leishmania* parasites. Therefore, an in vivo assessment of cytotoxicity and antileishmanial activity, as well as quantitative identification of secondary metabolites and evaluation of the activity of individual components against leishmaniasis, is very useful for formulating possible drugs.

## Acknowledgments

First of all, we thank the Department of Medical Parasitology, School of Biomedical and Laboratory Sciences, University of Gondar. Next, we acknowledge the Leishmaniasis Research and Diagnostic Laboratory, Addis Ababa University.

## Author contributions

**Conceptualization:** Kassahun Misgana Worku, Dawit Araya, Habtie Tesfa, Eshetie Melese Birru, Asrat Hailu, Mulugeta Aemero.

**Data curation:** Kassahun Misgana Worku, Dawit Araya, Habtie Tesfa, Eshetie Melese Birru, Asrat Hailu, Mulugeta Aemero.

**Formal analysis:** Kassahun Misgana Worku, Dawit Araya, Habtie Tesfa, Eshetie Melese Birru, Asrat Hailu, Mulugeta Aemero.

**Investigation:** Kassahun Misgana Worku, Dawit Araya, Habtie Tesfa, Eshetie Melese Birru, Asrat Hailu, Mulugeta Aemero.

**Methodology:** Kassahun Misgana Worku, Dawit Araya, Habtie Tesfa, Eshetie Melese Birru, Asrat Hailu, Mulugeta Aemero.

**Project administration:** Kassahun Misgana Worku, Habtie Tesfa, Eshetie Melese Birru, Asrat Hailu, Mulugeta Aemero.

**Resources:** Kassahun Misgana Worku, Habtie Tesfa, Eshetie Melese Birru, Mulugeta Aemero, Asrat Hailu.

**Software:** Kassahun Misgana Worku, Habtie Tesfa, Eshetie Melese Birru, Asrat Hailu, Mulugeta Aemero.

**Supervision:** Kassahun Misgana Worku, Habtie Tesfa, Eshetie Melese Birru, Asrat Hailu, Mulugeta Aemero.

**Validation:** Kassahun Misgana Worku, Dawit Araya, Habtie Tesfa, Eshetie Melese Birru, Asrat Hailu.

**Visualization:** Kassahun Misgana Worku, Dawit Araya, Habtie Tesfa, Eshetie Melese Birru, Asrat Hailu, Mulugeta Aemero.

**Writing – original draft:** Kassahun Misgana Worku, Dawit Araya, Habtie Tesfa, Eshetie Melese Birru, Asrat Hailu, Mulugeta Aemero.

**Writing – review & editing:** Kassahun Misgana Worku, Habtie Tesfa, Eshetie Melese Birru, Asrat Hailu, Mulugeta Aemero.
